# Circulating pro- and anti-angiogenic factors in multi-stage liver disease and hepatocellular carcinoma progression

**DOI:** 10.1038/s41598-019-45537-w

**Published:** 2019-06-24

**Authors:** Yu Young Joo, Jeong Won Jang, Sung Won Lee, Sun Hong Yoo, Jung Hyun Kwon, Soon Woo Nam, Si Hyun Bae, Jong Young Choi, Seung Kew Yoon

**Affiliations:** 1 Department of Internal Medicine, College of Medicine, The Catholic University of Korea, Seoul, Republic of Korea; 2 The Catholic University Liver Research Center, Seoul, 06591 Republic of Korea

**Keywords:** Hepatocellular carcinoma, Liver diseases

## Abstract

To date, few studies have carried out a simultaneous determination of multiple pro- and anti-angiogenic factors during liver diseases progression. This study investigated the dynamic change in circulating angiogenic factors in multi-step carcinogenesis and hepatocellular carcinoma (HCC) progression. Serum levels of major pro-angiogenic [Vascular endothelial growth factor (VEGF), Basic fibroblast growth factor (b-FGF)] and anti-angiogenic [Thrombospondin-1 (TSP-1), Endostatin] factors were identified by enzyme-linked immunosorbent assay and correlated with liver diseases progression and outcomes of HCC patients undergoing transarterial chemo-embolization. A total of 240 patients (156 HCC, 37 cirrhosis and 47 chronic hepatitis) were enrolled in this study. While progressing from chronic hepatitis, cirrhosis to HCC, VEGF and b-FGF levels showed a significant change. Particularly, b-FGF yielded the highest AUROC value for a diagnosis of HCC and its distinction from other disease groups. A trend towards increasing VEGF levels was observed from Child-Pugh class A, B to C. VEGF and TSP-1 levels increased with the advance of cancer stage, with a remarkable increase in TSP-1 at an intermediate stage. Pretreatment levels of VEGF, TSP-1, and endostatin independently predicted the overall survival of patients. VEGF and TSP-1 levels correlated with progression-free survival. Our study demonstrated the dynamic angiogenic switch and the roles that individual pro- and anti-angiogenic factors contribute to carcinogenesis and HCC progression during the course of multi-step liver diseases. These imply the future possibility of testing pro- and anti-angiogenic panels as a diagnostic marker and a guide in decision-making about upcoming targeted therapies.

## Introduction

Angiogenesis is the process in which a sprout is created from the existing fine vessels and develops new blood vessels^1,2^. Angiogenesis occurs not only in physical conditions but also in pathology reactions, a classic example of cancer. This process has as a key role in tumor progression^1,2^. Studies are being performed on the clinical availability of vascular progressivity in solid cancers as a cancer outcome factor or a predictive factor for therapeutic response.

Hepatocellular carcinoma (HCC) is typically a hypervascular tumor and angiogenesis plays a pivotal role in its progression. Angiogenesis is influenced by the microenvironment and tightly regulated by the balance between pro- and anti-angiogenic factors^1^. The capacity to stimulate angiogenesis is acquired by shifting the balance between these stimulatory and inhibitory factors of angiogenesis towards pro-angiogenic factors^1^. There are many pro-angiogenic and anti-angiogenic factors that affect angiogenesis. Vascular Endothelial Growth Factor (VEGF) and Basic Fibroblast Growth Factor (b-FGF) are major stimulatory angiogenic factor, whereas Thrombospondin-1 (TSP-1) and Endostatin are major inhibitory factors of angiogenesis^1,3–5^.

Despite the importance of angiogenesis in HCC, its role in multistep hepatic carcinogenesis has been inadequately studied. Studies to date have often been limited to only one or two angiogenic factors, and have included only a few patients or groups to be investigated, or short-term follow-up. Information on the balance and dynamic changes in expression of pro and anti-angiogenic factors during the course of liver disease progression from chronic hepatitis, cirrhosis, to HCC has been almost lacking thus far. In this study, we investigated the dynamic balance between pro- and anti-angiogenic factors and the angiogenic switch in multi-step liver diseases and HCC progression, by the simultaneous measurement of circulating angiogenic factors.

## Results

### Patient characteristics

The clinical characteristics of the patients at the time of diagnosis of liver diseases are shown in Table 1. The study population consisted of a total of 240 patients who included 47 with chronic hepatitis, 26 with compensated cirrhosis, 11 with decompensated cirrhosis and 156 with HCC. Patients enrolled included 173 males and 67 females, aged 59.1 ± 11.4 years. Child-Pugh classification of patients placed 173 in class A, 50 in class B, and 17 in class C. For patients with HCC, the mean tumor size was 7.3 ± 4.8 cm and HBV infection (80.0%) was the predominant cause of HCC. Treatments of HCC included hepatectomy (n = 7), TACE (n = 116), combined TACE (n = 15), and best supportive care (n = 18).

**Table 1:** Baseline characteristics of patients.

	Chronic hepatitis (n = 47)	Comp LC (n = 26)	Decomp LC (n = 11)	HCC (n = 156)
Age (years)	50.2 ± 12.2	57.7 ± 0.1	54.2 ± 4.9	59.1 ± 11.4
Sex (M:F)	23: 24	16: 10	7: 4	127 : 29
AST (IU/L)	56.7 ± 90.6	50.4 ± 38.9	120.3 ± 130.2	99.9 ± 105.4
ALT (IU/L)	69.7 ± 136.7	47.5 ± 60.2	73.6 ± 79.5	56.3 ± 48.2
Total bilirubin (mg/dl)	1.1 ± 0.6	1.3 ± 0.4	6.5 ± 8.8	2.1 ± 3.3
Albumin (g/dl)	4.2 ± 0.4	3.7 ± 0.5	3.1 ± 0.5	3.5 ± 0.6
Prothrombin time (INR)	1.1 ± 0.1	1.2 ± 0.1	1.6 ± 0.4	1.9 ± 9.2
Etiology (n)				
HBV/HCV/Alcohol/others	42/3/1/1	24/2/0/0	11/0/0/0	115/11/15/15
Child-Pugh score	5.2 ± 0.6	5.5 ± 0.8	8.2 ± 2.4	6.3 ± 1.7
A/B/C	44/3/0	24/2/0	3/4/4	102/41/13
AFP (ng/ml)^†^	2.8, 3.1	8.3, 16.8	18.7, 63.5	100.1, 1869.1
Tumor size (cm)				7.3 ± 4.8
Total size (cm)				9.9 ± 7.3
Tumor number				2.4 ± 1.56
Portal vein thrombosis				53 : 103
Distant metastasis				34 : 122
VEGF (ng/ml)	234.2 ± 242.6	133.5 ± 115.3	172.4 ± 68.1	273.7 ± 286.2
b-FGF (pg/ml)	8.9 ± 2.8	9.2 ± 2.4	9.1 ± 4.4	20.5 ± 13.2
Thrombospondin-1 (ng/ml)	132.4 ± 87.2	91.1 ± 60.6	126.7 ± 89.7	113.0 ± 95.7
Endostatin (ng/ml)	1.1 ± 0.5	1.0 ± 0.4	1.0 ± 0.4	1.5 ± 1.5

Comp LC, compensated liver cirrhosis; Decomp LC, decompensated liver cirrhosis; HCC, hepatocellular carcinoma; AST, aspartate aminotransaminase; ALT, alanine aminotransferase; INR, international normalized ratio; HBV, hepatitis B virus; HCV, hepatitis C virus; AFP, a-fetoprotein; VEGF, vascular endothelial growth factor; b-FGF, basic fibroblast growth factor.

^†^Kruskal-wallis test.

### Levels of angiogenic factors in liver disease progression

**Figure 1:**
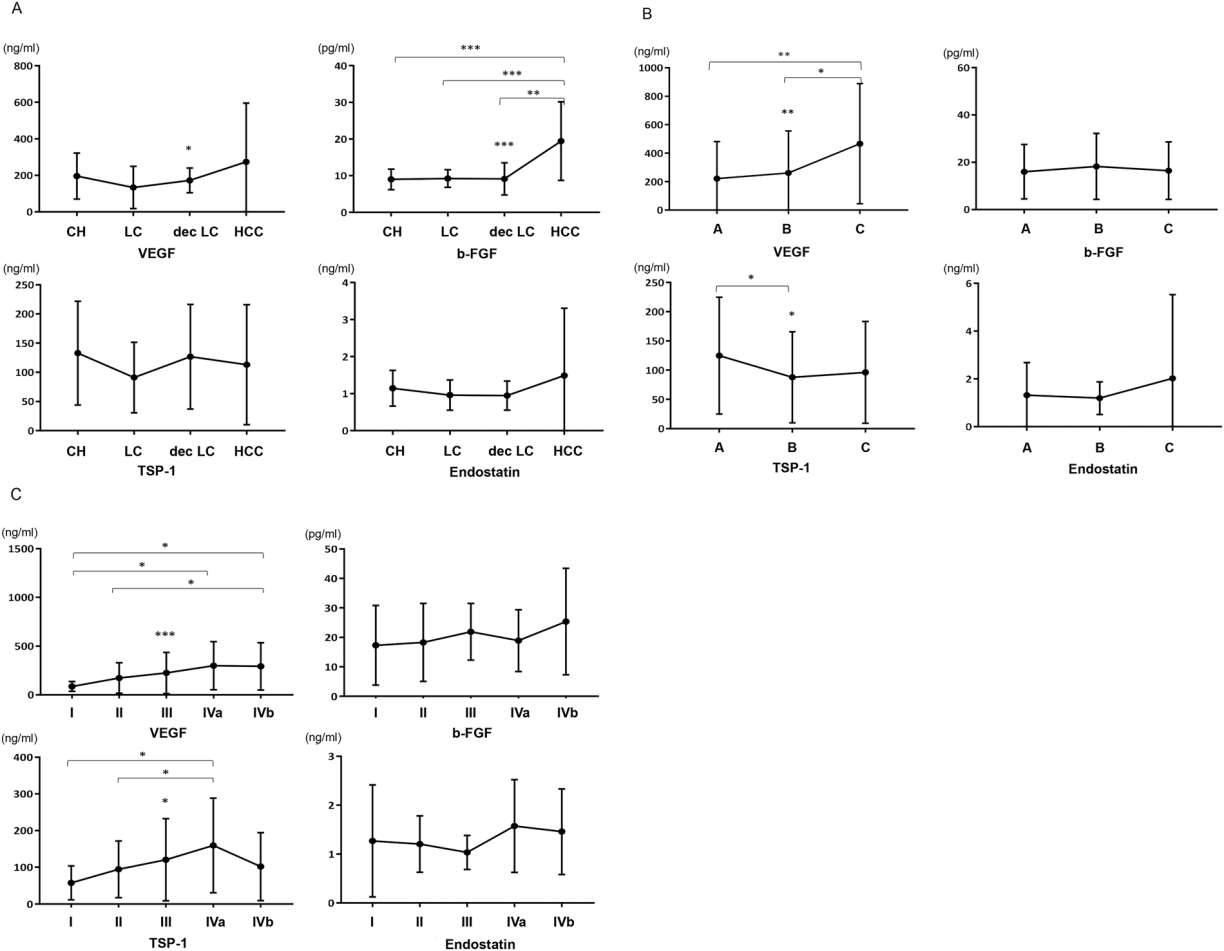
(**A**) Changes in serum VEGF, b-FGF, TSP-1, and endostatin levels across liver diseases of patients. (**B**) Serum levels of VEGF, b-FGF, TSP-1, and endostatin according to Child-Pugh class. (**C**) Differences in serum VEGF, b-FGF, TSP-1, and endostatin levels depending on modified UICC stage of HCC (*p < 0.05, **p < 0.01, ***p < *0.001*).

**Figure 2:**
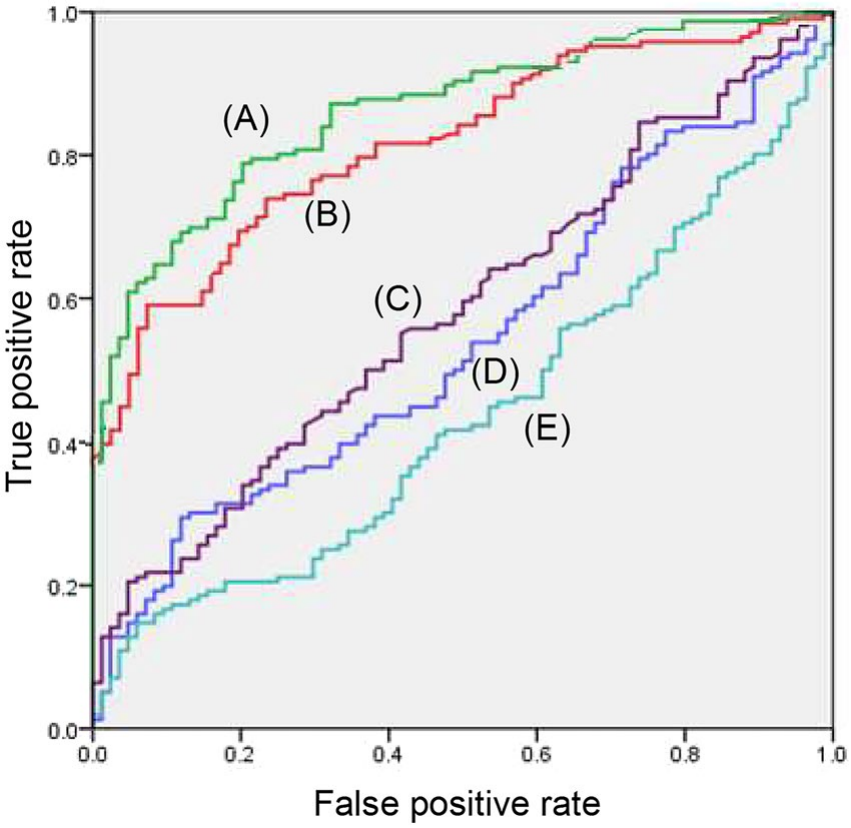
Comparison of the AUROC curves of AFP and angiogenic factors. (**A**) b-FGF exhibited a higher AUROC for diagnosing HCC than did other angiogenic factors. The AUROCs were 0.862, 0.819, 0.584, 0.543, and 0.440 for (**A**) b-FGF, (**B**) AFP, (**C**) endostatin, (**D**) VEGF, and (**E**) TSP-1, respectively.

Figure 1A shows changes in the angiogenic factors across disease progression from chronic hepatitis, cirrhosis, to HCC. Levels of VEGF (*p* = *0.045*) and b-FGF (*p* < *0.001*) made meaningful differences according to liver disease progression. Particularly, there was a dramatic rise in b-FGF levels in HCC compared to other liver diseases. To test the diagnostic performance of angiogenic factors for HCC, we compared the AUROC curves of AFP and angiogenic factors. As a result, b-FGF exhibited a higher AUROC for diagnosing HCC than did other angiogenic factors; that for b-FGF was higher than that of AFP [0.862 vs 0.819, respectively (Fig. 2)]. We compared the AUROC curves of angiogenic factors separately in the entire AFP-negative (≤20 ng/ml) and -positive (>20 ng/ml) population as well. In both groups, b-FGF had a higher AUROC value than that of other angiogenic factors and AFP (b-FGF = 0.858, Supplementary Fig. S1; b-FGF = 0.857, Supplementary Fig. S2).

When analyzed by Child-Pugh classification, VEGF levels increased from Child-Pugh class A, B, and to C (*p* = *0.004*), whereas TSP-1 levels decreased from class A to B or C [*p* = *0.036*; Fig. 1B)]. In patients with HCC, the levels of serum angiogenic factors differed depending on the tumor stage. VEGF levels displayed a gradual increase from stage I to IVb (*p* < *0.001*), whereas TSP-1 levels increased from stage I to IVa, but declined in stage IVb and thereafter (*p* = *0.016*). In addition, there were tendencies towards increasing b-FGF levels with the advancement of HCC stage (*p* = *0.159*) [Fig. 1C]. The overall findings indicate a dynamic change in pro- and anti-angiogenic factors across disease category, liver functional deterioration, and tumor stage over the course of natural progression of liver disease.

### Angiogenic factors in survival

**Figure 3:**
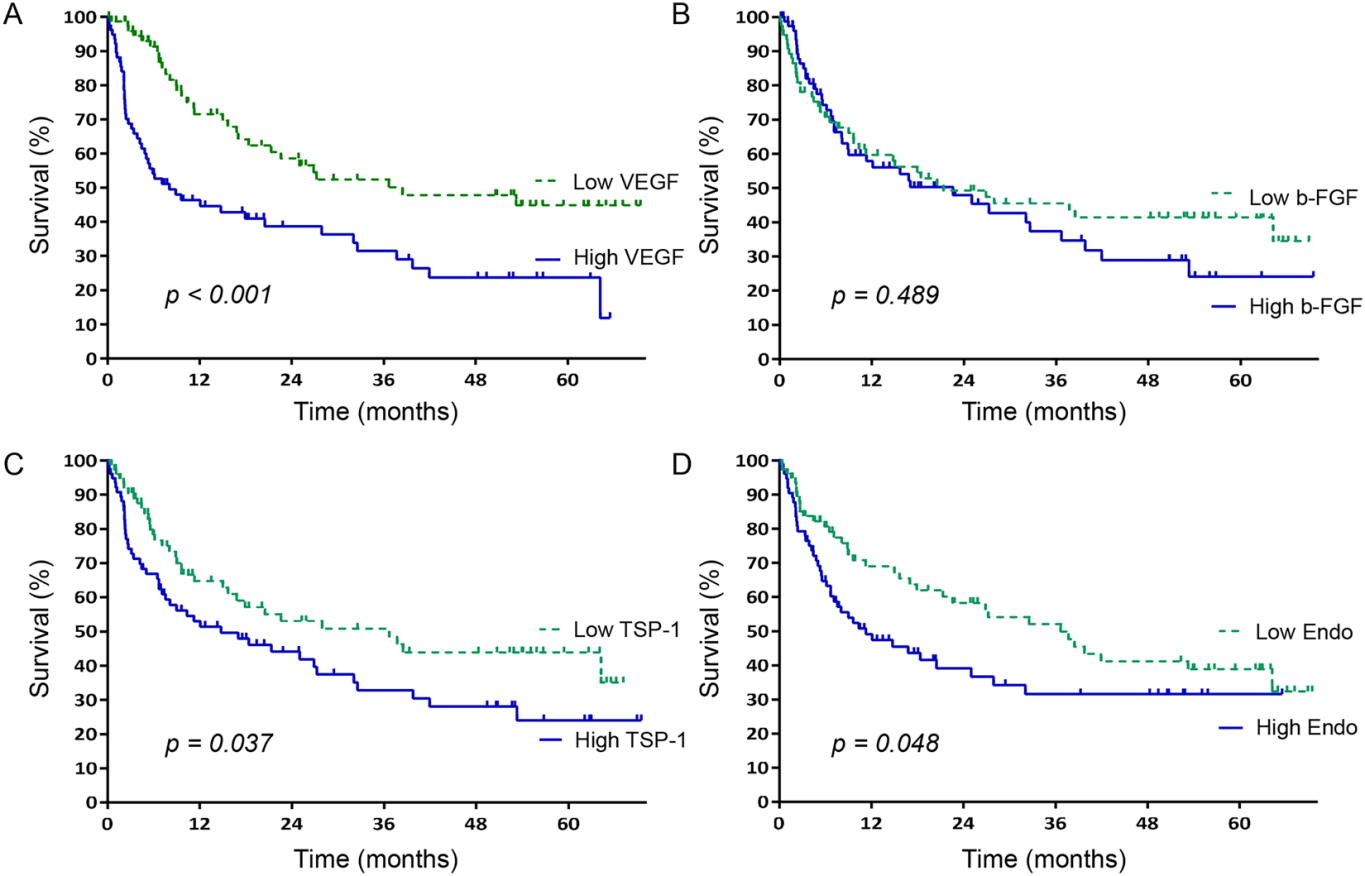
Kaplan–Meier curves of overall survival (**A**) in the high vs. low VEGF groups, (**B**) in the high vs. low b-FGF groups, (**C**) in the high vs. low TSP-I groups, and (**D**) in the high vs. low endostatin groups. High levels of VEGF, TSP-1, and endostatin were associated with shorter patient survival.

During the median follow-up time of 22.70 months (range 0.10–70.20), 87 (55.7%) patients died, 34 (21.8%) were lost to follow up, 11 (7.1%) were transferred to another hospital, and 24 (15.4%) were still alive until the last follow-up. The leading causes of deaths were HCC progression 43(49.4%), followed by hepatic deterioration 37 (42.5%) pneumonia 2 (2.3%), sepsis 1 (1.1%), cerebral hemorrhage 1 (1.1%), suicide 1 (1.1%), accidental death 1 (1.1%) and radiation pneumonitis 1 (1.1%). The 1-, 3-, and 5-year overall survival rates of the patients were 55.9%, 33.2%, and 17.6% respectively, with a median survival time of 17.03 months. As depicted in Fig. 3, pretreatment levels of angiogenic factors were associated with overall survival. Survival of patients with high VEGF (>150.59 ng/ml) levels was significantly lower than that of those with low VEGF levels (≤150.59 ng/ml) (8.07 vs. 38.47 months, *p* < *0.001*). Similarly, the overall survival of patients with high levels of TSP-1 and endostatin was significantly lower than that of those with low levels of them. The median survival times of patients with high (>81.98 ng/ml) and low TSP-1 levels were 14.77 and 36.67 months, respectively (*p* = *0.037*), while the corresponding times of those with high (>1.03 ng/ml) and low endostatin levels were 11.30 and 36.67 months, respectively (*p* = *0.048*).

**Figure 4:**
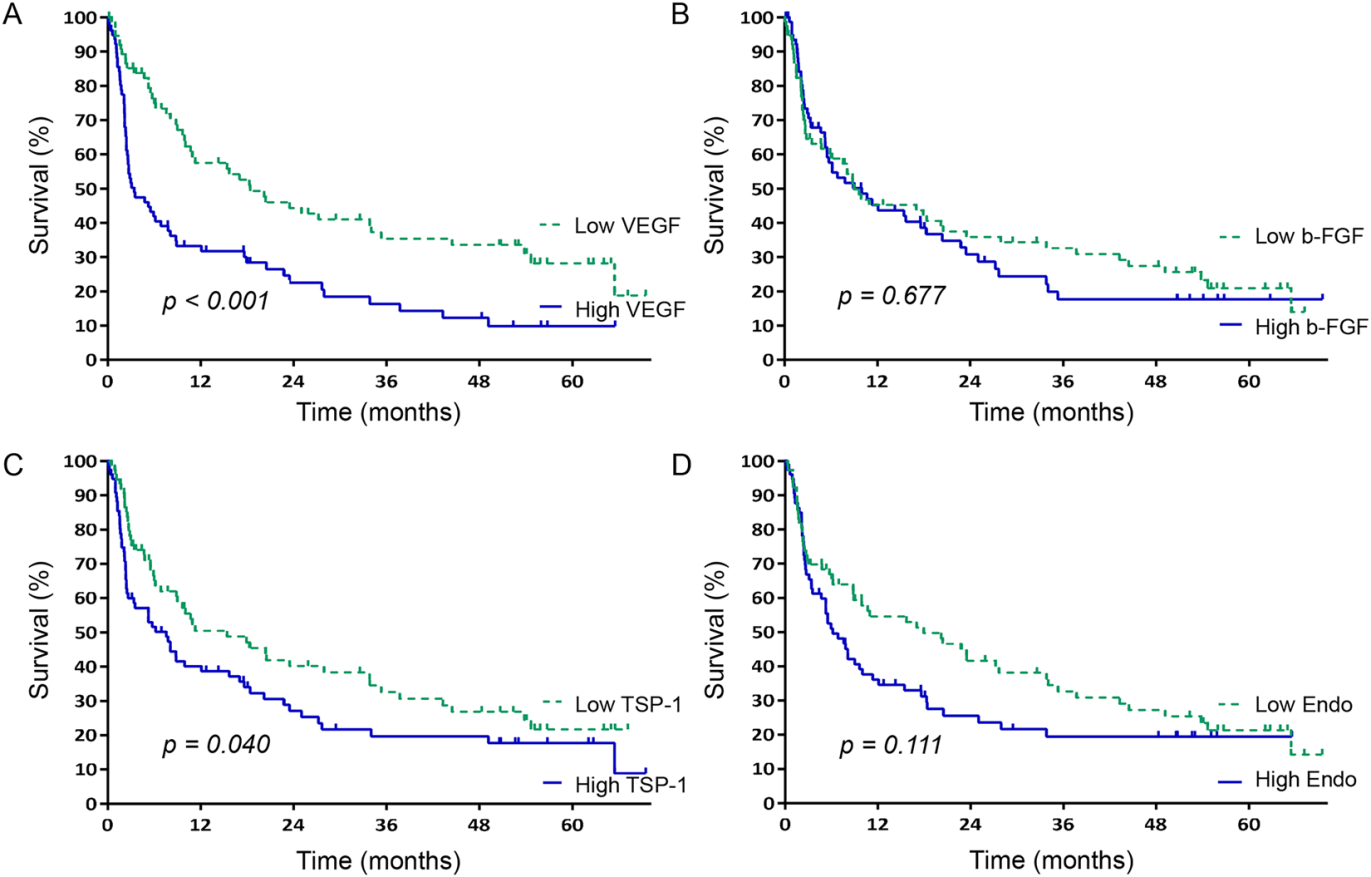
Kaplan–Meier curves of progression-free survival (**A**) in the high vs. low VEGF groups, (**B**) in the high vs. low b-FGF groups, (**C**) in the high vs. low TSP-I groups, and (**D**) in the high vs. low endostatin groups. High levels of VEGF and TSP-1 were associated with shorter progression-free survival.

For progression-free survival, pretreatment levels of VEGF and TSP-1 were identified to be the major influential factors (Fig. 4). Patients with high-levels of VEGF had significantly worse progression-free survival than those with less VEGF (3.43 vs. 18.40 months, respectively; *p* < *0.001*). Likewise, patients with high-levels of TSP-1 showed significantly worse progression-free survival than those with less TSP-1 (7.57 vs. 15.40 months, respectively; *p* = *0.040*). The overall findings indicate that the levels of VEGF, TSP-1, and endostatin affect the clinical outcomes of patients with HCC.

### Prognostic factors

To examine factors that affected patient survival, the nine clinical variables of our interest and four angiogenic factors were included in the analysis (Table 2). Univariate analysis showed that Child-Pugh class (*p* < *0.001*), AFP level >200 ng/ml (*p* = *0.011*), tumor size > 5 cm (*p* < *0.001*), portal vein thrombosis (*p* < *0.001*), distant metastasis (*p* < *0.001*), and the level of VEGF (*p* < *0.001*) and TSP-1 (*p* = 0.039) were significantly associated with worse survival of patients with HCC. In multivariate analysis, angiogenic factors together with baseline clinical and tumor factors remained significant. The levels of VEGF (odds ratio [OR], 1.78; 95% confidence interval [CI], 1.09–2.91), TSP-1 (OR, 2.38; 95% CI, 1.46–3.87) and Endostatin (OR, 1.70; 95% CI, 1.03–2.82) were independently predictive of poor survival among patients with HCC (Table 2).

**Table 2:** Analysis of prognostic factors for HCC.

Characteristics	Category	Overall survival		Univariate	Multivariate		
		1 year (%)	3 years (%)	*p*	OR	95% CI	*p*
Age	>50 yrs	62.2	43.3	0.110			
	≤50 yrs	45.3	26.9				
Sex	Male	55.8	41.0	0.327			
	Female	68.4	37.2				
Etiology	Viral	55.6	39.7	0.417			
	Non-viral	68.3	37.6				
Child-Pugh class	A	70.3	50.2	<0.001	0.21	0.12–0.36	<0.001
	B/C	27.7	20.2				
AFP level	>200 ng/ml	43.6	25.0	0.011	0.92	0.57–1.48	0.738
	≤200 ng/ml	67.7	49.3				
Tumor size	>5 cm	32.7	18.2	<0.001	2.16	1.10–4.23	0.024
	≤5 cm	88.0	64.3				
Tumor number	Multiple	38.8	25.3	<0.001	1.40	0.79–2.46	0.239
	Single	82.8	57.9				
PVT	Absence	79.4	58.0	<0.001	0.25	0.13–0.49	<0.001
	Presence	12.6	2.5				
Distant metastasis	Absence	66.5	50.1	<0.001	0.48	0.27–0.85	0.013
	Presence	26.2	0				
VEGF^†^	High	44.6	29.0	<0.001	1.77	1.08–2.90	0.022
	Low	71.5	50.1				
b-FGF^†^	High	56.1	34.7	0.489			
	Low	59.7	43.5				
TSP-1^†^	High	51.4	30.5	0.039	2.37	1.45–3.87	0.001
	Low	64.8	48.5				
Endostatin^†^	High	47.4	31.6	0.050	1.70	1.02–2.82	0.040
	Low	69.0	49.9				

HCC, hepatocellular carcinoma; OR, odds ratio; CI, confidence interval; AFP, a-fetoprotein; PVT, portal vein thrombosis; VEGF, vascular endothelial growth factor; b-FGF, basic fibroblast growth factor; TSP-I, thrombospondin-1.

^†^Cut-off levels: 150.59 ng/ml for VEGF, 12.13 pg/ml for b-FGF, 81.98 ng/ml for TSP-1, 1.03 ng/ml for Endostatin.

## Discussion

HCC is one of the most lethal malignant tumors worldwide. With only a few cases amenable to curative treatments, such as hepatectomy or liver transplantation, TACE is commonly used as part of a palliative treatment^6^. Thus, there exists a need to look for novel therapeutic strategies for the amelioration of HCC prognosis. Since HCC is a hypervascular tumor, involving a rich network of blood vessels and angiogenic components in the growth and metastasis of tumor, the inhibition of angiogenesis by effective therapies could hold tremendous therapeutic potential for better management of HCC^5,7^. With simultaneous measurement of multiple pro- and anti-angiogenic factors across a multistep process of liver carcinogenesis, we herein delineated the roles that each angiogenic factor plays in the various spectra of liver diseases, offering the potential of these factors as biomarkers for a multitude of opportunities for therapeutic intervention in HCC.

VEGF is the most widely studied pro-angiogenic factor and is shown to play a central role in tumorigenesis, progression, and metastasis through the process of angiogenesis in a variety of cancers^1,3–5,8^. In our study, VEGF was associated with the most clinical measures such as HCC development, progression, and survival. In addition, we could identify the association between serum VEGF levels and the worsening of liver function, as measured by the Child-Pugh classes. This is in line with prior studies showing the higher levels of VEGF in patients with variceal bleeding, portal hypertension, or fibrogenesis^3,7^. Thus, given that cancer progression and functional deterioration are among the two main causes of mortality in patients with HCC, our findings confirm again the important role of VEGF as a key driver of angiogenesis involving the entire process of liver disease progression.

TSP-1 has a dual effect on angiogenesis. As an anti-angiogenic factor, it reportedly provides good outcomes in colon and bladder cancer by inhibiting angiogenesis^9,10^. On the other hand, it has also been associated with poor outcomes in breast and gastric cancer, leading to tumor invasion^11,12^. In this study, serum TSP-1 levels decreased with deterioration in liver function, but increased with HCC progression. Although previously known as a representative anti-angiogenic factor, we observed that TSP-1 appeared to be as a poor prognostic indicator for overall and progression-free survival, correlating with advancing stages of HCC. This intriguing finding could be explained by the angiogenic switch, which promotes angiogenesis^1–3,5,8^. During early stages, the level of stromal TSP-1 is high enough to inhibit neovascularization and delay tumor growth. With disease progression, however, the prolonged hypoxia and an increase in VEGF secretion from tumor cells override the effect of TSP-1, therefore stimulating angiogenesis. In this later stage, TSP-1 functions as an adhesive protein or a modulator of extracellular proteases to promote tumor invasion^13^.

Besides their association with disease category as well as tumor characteristics, pretreatment levels of angiogenic factors played a role as a prognostic indicator for HCC survival. Particularly, VEGF, TSP-1, and endostatin levels independently predicted overall survival of HCC patients. Endostatin is a well-known anti-angiogenic factor; it inhibits metastasis and tumor growth in cancer^4^. As in cases of TSP-1, the higher level of endostatin correlated with low overall survival. It seems likely to increase with a background that is caused by an angiogenic switch, whereas other studies show that the behavior of endostatin as an anti-angiogenic factor depends on its concentration^14,15^. It is speculated that the anti-angiogenic and antitumor efficacy of endostatin is biphasic and too high or too low levels of circulating endostatin are inactive^14,15^. If tumors produce both stimulators and inhibitors of angiogenesis, the stimulators (VEGF, b-FGF) could accumulate in excess of inhibitors within an angiogenic tumor. In circulation, however, the ratio would be reversed. Angiogenesis inhibitors would increase in correlation to stimulators, because of rapid clearance of stimulators from the blood^15^.

Of note is the distinct change in b-FGF levels across the multi-step progression of liver disease. Particularly, the AUROC curve of b-FGF appeared to be higher than that of AFP (0.862 vs. 0.819), suggesting its potential as a diagnostic marker for HCC. The diagnostic performance of b-FGF was more apparent in the AFP-negative (≤20 ng/ml) group. Indeed, we identified that the level of b-FGF remained almost unchanged prior to HCC and then significantly increased at HCC development, which was not observed in other angiogenic factors. This indicates that apart from its pro-angiogenic property like VEGF, b-FGF might specifically play a fundamental role in the step of HCC development. Given the recent advances in anti-angiogenic therapy targeting the b-FGF pathway, the cancer-promoting role unique to b-FGF together with its function as a prognostic indicator deserves further research and investigation^16^.

In this study, we attempted to simultaneously measure multiple pro- and anti-angiogenic factors in patients with various liver diseases. We found out that those factors in general displayed a trend towards increasing levels of hepatitis, cirrhosis, to HCC, with the most significant increase in VEGF and b-FGF levels. It is important to understand that these various dynamic changes occur in the multi-step liver diseases and HCC progression.

This study has several limitations. We analyzed only the circulating levels of angiogenic factors, but did not do tissue analysis. Thus, we cannot comment on a correlation between serum and tissue factors. The cut-offs for high- versus low-levels of angiogenic factors have yet to be defined. Although b-FGF showed good diagnostic performance for HCC, whether b-FGF can be exactly superior to AFP should be evaluated in further studies involving other patient groups with different characteristics. Last, the study subjects were only patients receiving TACE. Thus, our results might not be generalizable to patients undergoing different treatments. Nevertheless, our work is meaningful in that it identified pro- and anti- angiogenic factors simultaneously and their dynamic interplay over the natural course of liver disease advancement, since no studies to date had made such observations recruiting various liver diseases.

In conclusion, our study demonstrates that there are distinct changes in the levels of each pro- and anti-angiogenic factor during the multi-stage disease progression from hepatitis, cirrhosis, to HCC. The dynamic nature and interplay between the individual pro- and anti- angiogenic factors may contribute to liver carcinogenesis and HCC progression. Based on our study, an increase in VEGF is involved across the entire process of liver disease progression prior to and after HCC development, and b-FGF may significantly contribute to the major step of HCC development. Moreover, circulating VEGF, TSP-1 and endostatin independently predict overall survival of HCC patients. These results suggest a future possibility of testing pro- and anti-angiogenic panels as a diagnostic marker as well as a guide for decision-making about upcoming targeted therapies.

## Materials and Methods

### Patients

The study population was made up of 240 patients with blood samples who were followed at a liver unit at the Catholic University of Korea between November 2009 and December 2012. The 240 enrolled patients included 156 with newly diagnosed HCC who underwent TACE (transarterial chemoembolization) or best supportive care, and 84 with non-HCC liver disease (37 with liver cirrhosis and 47 with chronic hepatitis).

Liver cirrhosis (LC) was diagnosed when radiologic findings revealed cirrhosis features such as an inhomogeneous hepatic surface or portal hypertension like splenomegaly. Decompensated LC was defined as the presence of complications, such as ascites, variceal bleeding, peritonitis, hepatic encephalopathy or hepatorenal syndrome. The diagnosis of HCC was made by histological evidence or with typical radiological findings suggestive of HCC in computed tomography (CT) or magnetic resonance imaging (MRI)^17,18^. The stage of HCC was diagnosed according to the modified International Union Against Cancer (UICC) stage system- Korean NCCN guideline^17,18^.

Patients were followed up until death or the last day of follow-up (December 31, 2015). Each patient provided informed consent to participate in the study. This study was approved by the Ethics Committees of The Catholic University of Korea in accordance with the 1975 Declaration of Helsinki.

### Measurement of angiogenic factors

Angiogenic factors tested included major pro-angiogenic (VEGF, b-FGF) and anti-angiogenic (TSP-1, Endostatin) factors. Serum concentrations of VEGF, b-FGF, TSP-1, and endostatin were measured by enzyme-linked immunosorbent assay (Quantikine, R&D Systems, Minneapolis, MN, USA). Sampling of serum was obtained from the study subjects at the diagnosis of liver disease and stored in a freezer at −20 °C

### Treatment

Patients were offered transarterial therapy of intra-arterial doxorubicin (50 mg) with embolization or combined epirubicin (50 mg) and cisplatin (60 mg) in a mixture of lipiodol (5–10 ml) without gelfoam embolization, based on tumor stages^17,18^. The transarterial therapy was repeated at 6 to -8 week intervals by the general treatment protocol until the achievement of complete response defined as radiological disappearance or complete necrosis of the viable tumor on two consecutive occasions. Abdominal CT scan and/or MRI imaging with AFP level were checked every 2 to -3 months for response assessment during and after TACE. Treatment response was assessed using the modified Response Evaluation Criteria in Solid Tumors criteria (mRECIST criteria)^19^.

### Statistical analysis

Data were expressed the mean ± S.D. or median (range). The results were analyzed using the Student t test, Mann-Whitney U test, Kruskal-Wallis ANOVA (analysis of variance), and the Chi-square test, as appropriate. In our study, the median level of each angiogenic factor was considered to be the cut-off value for analysis. Diagnostic ability of each angiogenic factor for HCC was assessed and compared by a receiver operating characteristic (ROC) curve. Univariate and multivariate analyses with the Cox proportional hazard model were used to identify risk factors for overall survival. Survival time was defined as the duration from the diagnosis until death or follow-up endpoint, and progression-free survival from the diagnosis until progressive disease defined by mRECIST criteria. Overall and progression-free survival of the patients was calculated using the Kaplan-Meier method and compared by log-rank test. A p-value less than 0.05 was considered to be statistically significant. Statistical analysis was performed using IBM SPSS statistics 24 (SPSS Inc. Chicago, Illinois, USA).

## Supplementary information

Supplementary figures
