# Supplementary material for: Circulating pro- and anti-angiogenic factors in multi-stage liver disease and hepatocellular carcinoma progression
Source: Sci Rep. 2019 Jun 24;9:9137. doi: 10.1038/s41598-019-45537-w (PMC6591389; doi:10.1038/s41598-019-45537-w)
Supplement: Supplementary file 1 — Supplementary figures [file 41598_2019_45537_MOESM1_ESM.docx]

Original Article

**Circulating pro- and anti-angiogenic factors in multi-stage liver disease and hepatocellular carcinoma progression**

Yu Young Joo^1,2^, Jeong Won Jang^*1,2^, Sung Won Lee^1,2^, Sun Hong Yoo^1,2^, Jung Hyun Kwon^1,2^, Soon Woo Nam^1,2^, Si Hyun Bae^1,2^, Jong Young Choi^1,2^, Seung Kew Yoon^1,2^

^1^Department of Internal Medicine, College of Medicine, The Catholic University of Korea, Seoul, Republic of Korea

^2^The Catholic University Liver Research Center, Seoul 06591, Republic of Korea

**Corresponding author**

Jeong Won Jang, M.D., Ph.D.

Division of Hepatology, Department of Internal Medicine, Seoul St. Mary's Hospital, College of Medicine, The Catholic University of Korea, 222 Banpo-daero, Seocho-gu, Seoul 06591,

Republic of Korea

Telephone: +82-2-2258-6015, Fax: +82-2-3481-4025, E-mail: [garden@catholic.ac.kr](mailto:garden@catholic.ac.kr)


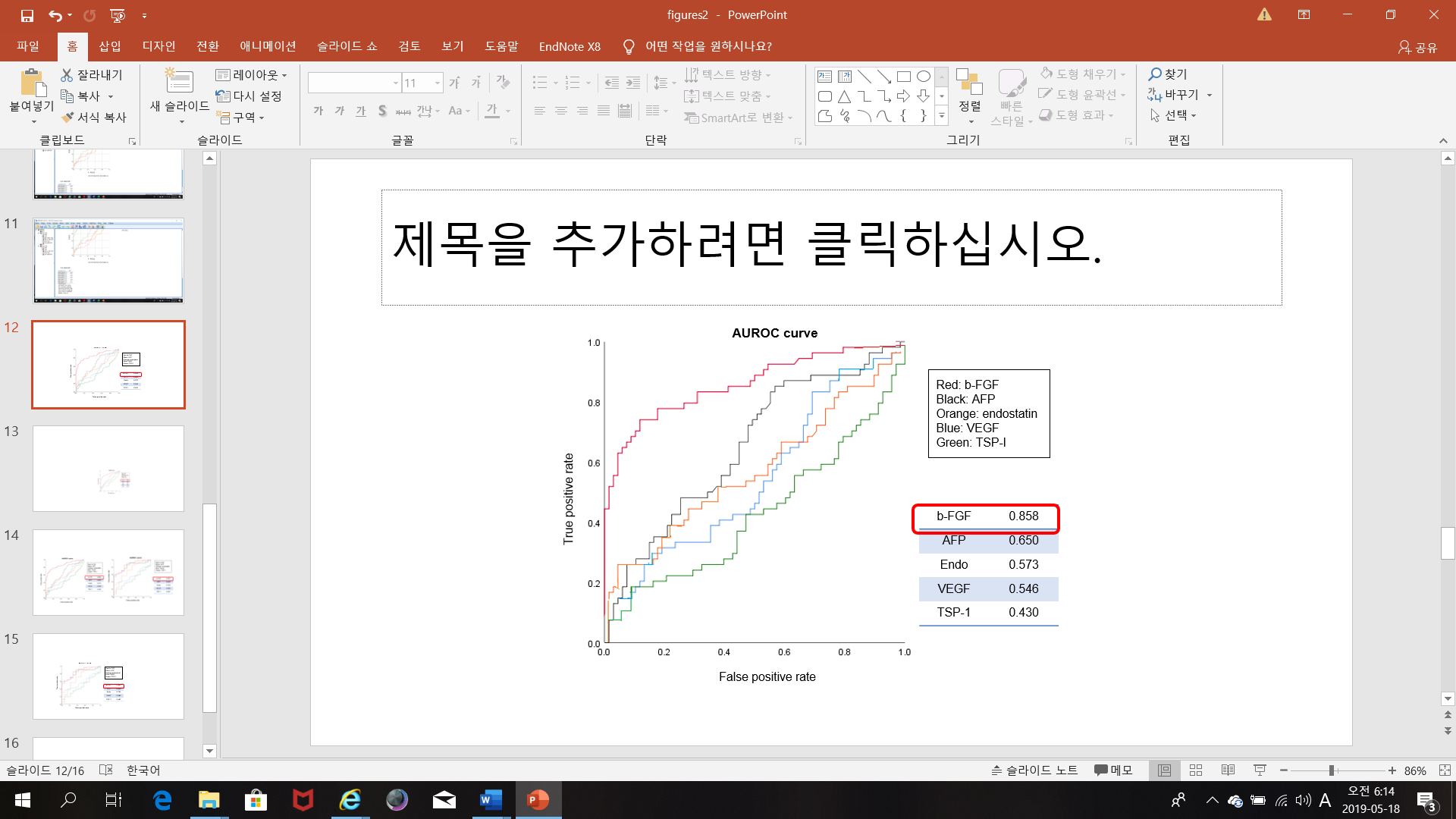


Supplementary Figure S1. Comparison of the AUROC curves of AFP and angiogenic factors in the AFP-negative population.


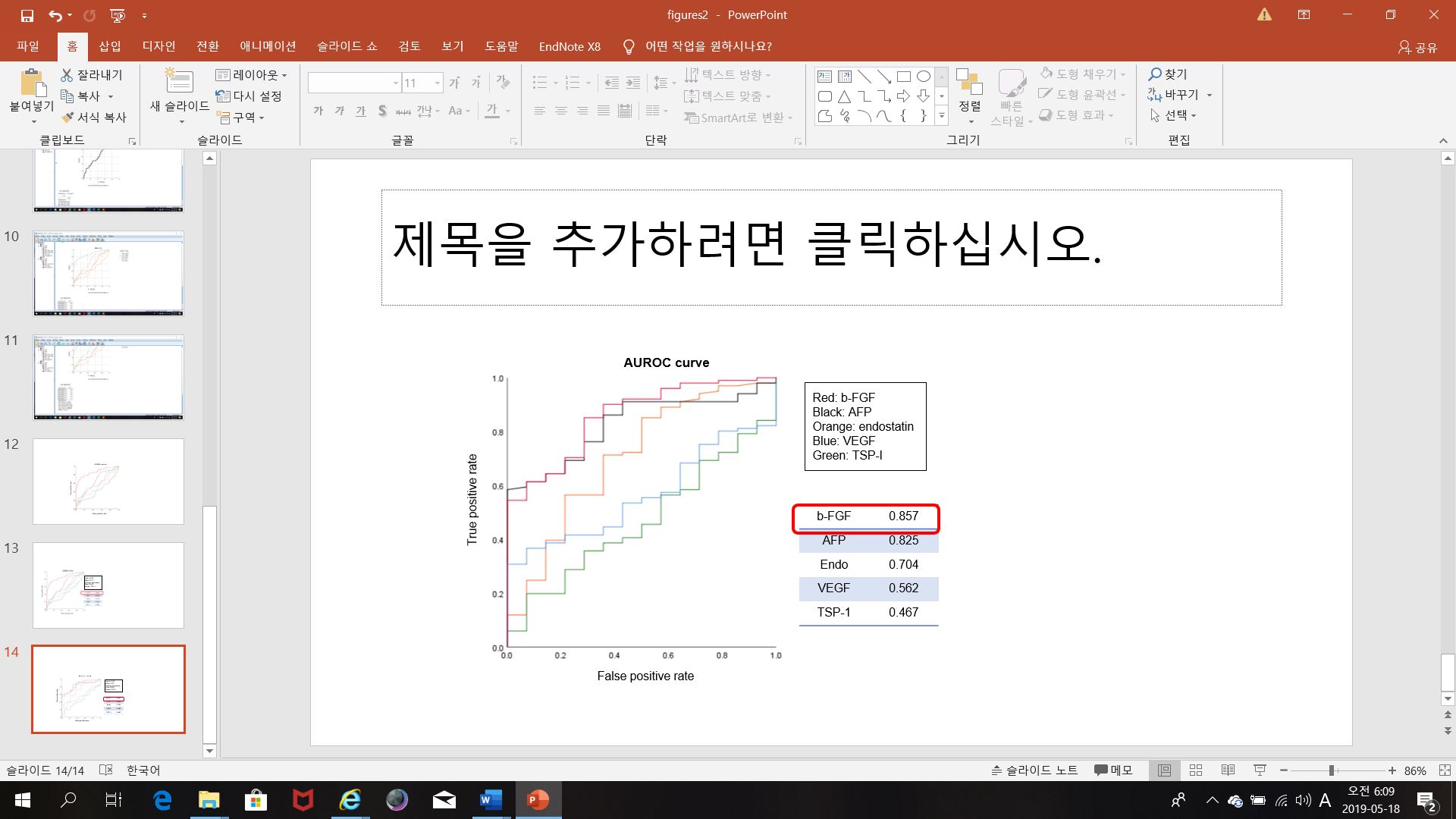


Supplementary Figure S2. Comparison of the AUROC curves of AFP and angiogenic factors in the AFP-positive population.
